# Contrasting Magnitude and Timing of Pulsed Aqueous Methylmercury Bioaccumulation across a Reservoir Food Web

**DOI:** 10.1021/acs.est.4c10719

**Published:** 2025-02-17

**Authors:** James J. Willacker, Collin A. Eagles-Smith, Austin K. Baldwin, Michael T. Tate, Brett A. Poulin, Jesse Naymik, David P. Krabbenhoft, Ralph Myers, James A. Chandler

**Affiliations:** † U.S. Geological Survey, Forest and Rangeland Ecosystem Science Center, 3200 SW Jefferson Way, Corvallis, Oregon 97331, United States; ‡ U.S. Geological Survey, Idaho Water Science Center, Boise, Idaho 83702, United States; § U.S. Geological Survey, Upper Midwest Water Science Center, 1 Gifford Pinchot Dr, Madison, Wisconsin 53726, United States; ∥ Department of Environmental Toxicology, University of California at Davis, Davis, California 95616, United States; ⊥ Idaho Power Company, 1221 West Idaho Street, Boise, Idaho 83702, United States

**Keywords:** bioaccumulation, bluegill, hypoxia, plankton, Snake River, smallmouth bass

## Abstract

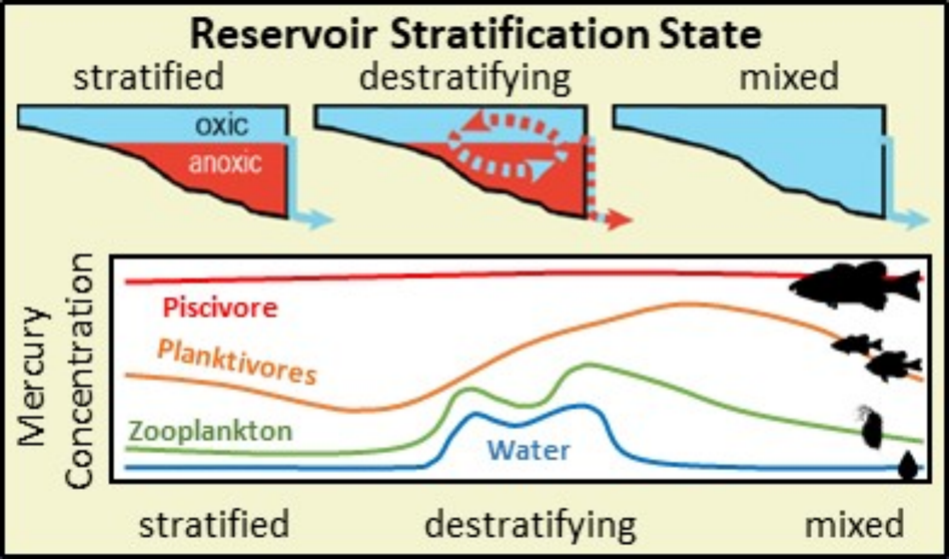

Water column hypoxia is a key process influencing methylmercury (MeHg) production and availability in waterbodies worldwide. During seasonal destratification, large, short-lived pulses of aqueous MeHg may be released into the subsequently mixed water column, but little is known about the fate of these pulses, particularly whether there are concomitant increases in MeHg uptake into aquatic food webs. We examined the magnitude and timing of MeHg uptake across several trophic guilds relative to the reservoir stratification status using biweekly mercury data from water, zooplankton, and fish (Bluegill, *Lepomis macrochirus* and Smallmouth Bass, *Micropterus dolomieu*). Zooplankton MeHg concentrations increased by up to 250% during destratification, concurrent with increases in aqueous MeHg concentrations. Zooplankton and filter-passing MeHg concentrations were positively correlated when the reservoir was mixed (*R*^2^ = 0.95) and destratifying (*R*^2^ = 0.57) but not while the reservoir was stratified (*R*^2^ = 0.21). Mercury concentrations in adult bluegill and juveniles of both fish species increased 20–70% following destratification, with responses lagging 4–8 weeks behind those in water and zooplankton MeHg. Mercury concentrations in piscivorous adult bass varied little over the course of the study. Our findings demonstrate the responsiveness of reservoir food webs to pulses in MeHg availability, suggesting that these pulses could play an important role in biotic MeHg exposure within and downstream of reservoirs.

## Abstract 2

Methylmercury is redistributed during destratification and rapidly incorporated into aquatic food webs, accumulating in zooplankton within 2 weeks and in planktivorous fishes in 4 to 8 weeks.

## 1 Introduction

Freshwater ecosystems play an important role in the cycling of environmental contaminants, particularly toxic methylmercury (MeHg), which is produced in aquatic environments under specific biogeochemical conditions.^1,2^ However, these ecosystems are also among the most sensitive to exploitation and environmental change, responding to shifts in climate, hydrology, habitat, land use, and chemical inputs, among many other factors.^3,4^ Often, these seemingly disparate influences on aquatic habitats have unforeseen consequences for other ecosystem properties, complicating identification of the drivers and processes modulating ecosystem responses or anticipation of their ultimate effects. Climate change, in particular, has resulted in a wide array of cascading effects on aquatic ecosystems,^5,6^ among which is a global increase in the occurrence and magnitude of low dissolved oxygen (DO) conditions,^7−11^ which in-turn has important implications for a variety of biogeochemical processes,^12^ including the production and fate of MeHg.^13−15^

DO plays a critical role in regulating key biological, chemical, and ecological processes in aquatic ecosystems.^16^ Hypoxic water conditions, when DO concentrations are less than 2 mg/L, typically result from organic matter accumulation and subsequent decomposition, during which microbial metabolism consumes oxygen.^17^ Warmer surface water temperatures,^18^ eutrophication,^19^ and shifts in algal community composition^20^ have increased aquatic primary productivity as well as the occurrence of episodic blooms that generate large quantities of organic matter over very short time periods.^21^ Decomposition of the resulting organic matter is a key process driving increased hypoxic conditions in aquatic ecosystems ranging from small inland lakes and rivers to the world’s oceans.^7,8^ Thus, far, research examining the consequences of more frequent and severe hypoxia in aquatic habitats has largely focused on changes in greenhouse gas emissions,^22^ nutrient cycling,^23^ or lethal effects on aquatic biota,^24^ while effects on contaminants such as MeHg have received relatively little attention.

Exposure to MeHg results in substantial impairment to human and wildlife health globally and is the leading cause of fish consumption advisories in the Unites States.^25,26^ While direct anthropogenic releases of MeHg to the environment are limited, MeHg concentrations in aquatic biota often exceed benchmarks associated with developmental or reproductive effects.^27^ This seeming paradox is because inorganic mercury (IHg) species, which are less toxic but widely emitted to the atmosphere and surface waters,^25,28^ can be readily converted to MeHg by anaerobic microbes in locations with favorable biogeochemical conditions, foremost among which is reduced DO concentrations.^1,2^ Hypoxic and anoxic (i.e., DO = 0 mg/L) conditions in aquatic sediments or water columns provide favorable redox conditions for MeHg production,^29,30^ while associated organic matter transports IHg^15^ and provides a key natural substrate for diverse microbial organisms capable of methylating IHg.^30^ When hypoxia is associated with thermal stratification, as is often the case,^9,10^ MeHg can build-up in the metalimnion and hypolimnion and be subsequently redistributed through the water column during destratification.^14,31,32^ The incorporation of this MeHg pulse into food webs has been documented;^31−34^ however, the rate of MeHg uptake from these pulses and subsequent redistribution through food webs is not well understood. This represents a significant knowledge gap since this information is important for predicting and interpreting the response of biotic MeHg concentrations to both local (e.g., nutrient reductions) and global (e.g., Minamata Convention on Mercury) management actions.^27,35,36^

Riverine impoundments (i.e., reservoirs) are particularly vulnerable to both climate driven hypoxia and MeHg impairment.^7,37,38^ Decomposition of terrestrial vegetation following initial impoundment or contemporary water level fluctuations is widely associated with increased biotic MeHg concentrations in reservoirs.^38−40^ Many impoundments are also predisposed to hypoxia as a result of settling watershed derived and in situ organic matter.^41,42^ As a result, water column MeHg production also contributes to, and in some cases may be the dominate cause of, elevated MeHg risk in many reservoirs.^43^ Furthermore, impounded habitats are also particularly important sources of human MeHg exposure due to their predominance across the landscape,^44−46^ highly productive fisheries,^47^ and frequent utilization by recreational and subsistence anglers.^48,49^ The management and remediation of reservoirs may benefit from an improved understanding of the linkages between reservoir hypoxia, MeHg formation, and MeHg uptake in the aquatic food web. Here, we report on the magnitude and timing of water column produced MeHg incorporation into a reservoir food web, a necessary first step for guiding MeHg remediation efforts in stratified reservoirs.

In this study, we quantify changes in aqueous MeHg during seasonal stratification and destratification of a reservoir and quantify the timeframes over which this MeHg is incorporated into specific components of the reservoir food web. Over a two-year period, water, zooplankton, three planktivorous fish groups, and a piscivorous fish were sampled at approximately two-week intervals to examine whether (1) the pulsed release of aqueous MeHg from the meta- and hypolimnion during destratification^15,50^ corresponded with increases in biotic MeHg concentrations, and (2) the rate at which pulsed MeHg is incorporated among various compartments of the aquatic food web.

## 2 Methods

### 2.1 Study Area

Brownlee Reservoir (Figure S1) is one of three impoundments on the Snake River along the Idaho-Oregon border (USA) comprising the Hells Canyon Complex. Although naturally semiarid, the watershed upstream of the complex has largely been converted to agriculture near the Snake River, which contributes nutrients and labile organic matter (e.g., allochthonous algae) to Brownlee Reservoir.^15,51^ The structure, watersheds, nutrient inputs, and biogeochemical processes in the Hells Canyon Complex, and Brownlee Reservoir in particular, have been characterized in detail elsewhere.^15,30,51−53^ Of particular relevance to the present study, settling and decomposition of watershed and reservoir-derived algal matter, coupled with Brownlee Reservoir’s depth (mean = 40 m, maximum = 91 m at full pool) and strong seasonal thermal stratification, result in large portions of the water column becoming hypoxic during the summer months.^51^ This stratification gradually erodes as the temperature of inflowing water cools, generally beginning in August, with the water column becoming completely mixed in December or January (Figure S2).^14,51,53^ Prior works have demonstrated the overwhelming importance of this seasonal hypoxia in determining MeHg production and aqueous MeHg concentrations in Brownlee Reservoir.^14,15,30,50^ These works examined a broad array of physical, chemical, and microbial data to assess the mechanisms underlying MeHg production in the reservoir and concluded that water column MeHg production is driven by obligate anerobic microbes^30^ and that the biogeochemical conditions that facilitate this phenomenon are dependent on the presence of hypoxic conditions.^15^ Large MeHg pools build up in the metalimnion and hypolimnion during stratification and are subsequently redistributed through the water column and then exported to the downstream reservoirs and Snake River during destratification.^14,15,30,50^ For the current study, we sampled the furthest downstream 6 km of Brownlee Reservoir (Figure S1), which encompasses the deepest and strongest stratifying portion of the reservoir.

### 2.2 Sample Collection, Processing, and Chemical Analyses

We collected water, zooplankton, and two species of fishes from Brownlee Reservoir approximately biweekly between June 2018 and March 2020. Detailed water collection methods are provided elsewhere,^14^ but consisted of depth-integrated samples collected at the thalweg of Brownlee Reservoir’s outflow from a bridge using a DH-95 sampler and Teflon bottle and nozzle. This location was selected because the outflow of Brownlee Reservoir represents a well-mixed sample of Brownlee Reservoir water drawn from the upper 25–40 m (depending on water elevation and flow conditions) of the water column^51,53^ and water could be collected throughout the year, even when depth-integrated water sampling of the reservoir water column was impractical or impossible. Within 24 h of collection, water samples were filtered through 0.7 μm pore size quartz fiber filters (QFF; precombusted to 550 °C) into precleaned Teflon bottles and then acidified to 1% volume-to-volume with ultraclean hydrochloric acid. We only present results from filtered water because prior work in this system has shown that aqueous filter-passing MeHg concentrations are most directly linked to in situ MeHg production and uptake into the reservoir food web.^14,15,30,50^

We collected bulk plankton from the upper 10 m of the water column using a 1 m diameter conical plankton net with a 153 μm mesh collection cup. Plankton were placed in PETG sample jars and kept on ice while in the field (≤10 h). Immediately following collection events, we rinsed samples through a 500 μm stainless steel mesh sieve to separate large-bodied zooplankton ≥500 μm, the size primarily targeted by most fishes, from bulk plankton. Microscopy indicated these samples were dominated by large Cladocera (50–99%, median = 84%), particularly *Daphnia galeata mendotae*. Zooplankton samples were frozen at −20 °C until processing for Hg analysis. During some sampling events (*n* = 26) we were not able to collect zooplankton directly from the reservoir. For these events we used the regression between MeHg concentrations in zooplankton samples concurrently collected from the reservoir and the outflow of Brownlee Reservoir (*n* = 14, *R*^2^ = 0.96, *p* < 0.001) during other events to estimate reservoir zooplankton concentrations.^14^ In the laboratory, we lyophilized frozen zooplankton samples at −40 °C, ground the dried samples to a fine powder using a ceramic mortar and pestle, and then stored the tissue in airtight scintillation vials until analysis.

Fishes (Bluegill, *Lepomis macrochirus*; and Smallmouth Bass, *Micropterus dolomieu*) were collected from Brownlee Reservoir via boat-based electrofishing. These species were selected because they are abundant, frequently targeted by anglers, and represent distinct trophic ecologies.^54^ At each sampling event, we targeted 10 individuals of each species with two size-classes per species: Bluegill ≤100 mm and bass ≤150 mm were collected to represent juveniles of each species, whereas fish ≥125 mm and ≥250 mm were targeted to represent adult Bluegill and bass, respectively.^54^ All fish were euthanized with buffered MS-222, placed in polyethylene bags, stored on ice while in the field (≤6 h), and then frozen at −20 °C until processing for Hg analysis. All fish collections were in accordance with Idaho Department of Fish and Game and Oregon Department of Fish and Wildlife permits, and animal care approvals issued to Idaho Power Company. In the laboratory, we measured the total length of each thawed fish to the nearest mm before removing an aliquot (ca. 5 g) of skinless axial muscle. Muscle samples were weighed to the nearest 0.0001 g, oven-dried at 50 °C (ca. 48 h), reweighed to determine percent moisture, and then homogenized to a fine powder using a ceramic mortar and pestle. Prior to analysis we stored dried-ground muscle samples in airtight scintillation vials.

Filtered water samples were analyzed for MeHg at the U.S. Geological Survey (USGS) Mercury Research Laboratory (MRL; Madison, WI) following USEPA Method 1630,^55^ with modifications by the MRL.^56^ The average daily detection limit for filter-passing aqueous MeHg was 0.013 ± 0.0012 ng/L, and we used the raw, uncensored data for any samples with concentrations less than the associated detection limit (*n* = 6).^57,58^ Quality assurance for aqueous MeHg (mean ± standard error) included analysis of field blanks (0.005 ± 0.0126 ng/L; *n* = 40) and sample replicates (*n* = 39; mean relative percent difference [RPD] = 24.4%). Quality assurance results are summarized in Table S1.

Zooplankton samples were analyzed for MeHg at the USGS Contaminant Ecology Research Laboratory (CERL; Corvallis, OR) using a MERX-M (Brooks Rand Instruments, Seattle, Washington) automated MeHg analyzer and following EPA method 1630.^55^ The average detection limit for tissue MeHg was 2.8 ± 0.01 ng/g dw. Quality assurance for tissue MeHg included analysis of calibration standards, certified reference materials (CRM; IAEA-452, IAEA-407), method blanks, and sample duplicates. Quality assurance results are summarized in Table S1.

We analyzed THg in fish muscle at CERL using a Nippon MA-3000 (Nippon Instrument Corporation, Osaka, Japan) Hg analyzer and following EPA method 7473 (U.S. Environmental Protection Agency, 2000). The average detection limit for THg was 0.002 ± 0.0001 mg/kg dw. Quality assurance for tissue THg included analysis of calibration standards, CRMs (DORM-4, TORT-3), method blanks, and sample duplicates. Quality assurance results are summarized in Table S1. We also analyzed a subset of fish samples spanning species, size-classes, sampling dates and THg concentrations for MeHg at CERL using the methods outlined above and calculated the percentage of THg represented by MeHg for this subset of fish (Bluegill: 96.8 ± 1.8%, *n* = 43; Smallmouth Bass: 88.5 ± 1.1%, *n* = 45) to confirm THg concentrations were an effective proxy for MeHg concentrations in these species.

### 2.3 Statistical Analyses

We analyzed all tissues dry and present concentrations on a dry-weight basis. All Hg data were natural-log transformed to meet the assumptions of normality and homogeneity of variance. We performed statistical analyses in JMP^59^ and unless otherwise noted, Hg data are presented as least-squares or geometric means with standard errors estimated using the delta method.^60^

We calculated size standardized THg concentrations for fish because raw THg concentrations were correlated with total length (Figure S3). Specifically, for each species and size-class, we standardized THg concentrations to the median length (juvenile Bluegill = 72 mm, adult Bluegill = 131 mm; juvenile bass = 89 mm, adult bass = 290 mm) using species-specific THg-length regressions (incorporating the entire sampled size range of each species) and the individual-specific residuals.^61^ We size standardized juveniles and adults of each species separately because MeHg bioaccumulation is often influenced by biological, ecological, and habitat differences among age-classes.^34^ Size standardized THg concentrations were used in all subsequent analyses.

We used regression with Pearson’s correlation coefficients to assess the relationships between paired fish, plankton, and water Hg concentrations. We also evaluated whether there were temporal lags in the relationships between filter-passing aqueous MeHg and zooplankton MeHg and between zooplankton MeHg and fish THg, using cross-correlation analysis.^62^ To examine the role of stratification status on Hg bioaccumulation, we classified the stratification status during each sampling event as stratified/stratifying, destratifying, or mixed determined by Baldwin et al.^14^ during the period of sampling for the current study. Differences in lag-adjusted Hg concentrations among stratification categories were tested using matrix-specific analysis of variance (ANOVA) with Tukey Honestly Significant Difference (HSD) *post hoc* multiple comparisons. Differences in aqueous MeHg–zooplankton MeHg and zooplankton MeHg–fish THg relationships among stratification statuses was tested using general linear models with stratification status as a fixed effect, either aqueous MeHg (for plankton model) or zooplankton MeHg (for fish models) as a covariate, and the interaction between stratification status and the covariate.

## 3 Results and Discussion

### 3.1 Seasonal Variation in Hg Concentrations

**Figure 1:**
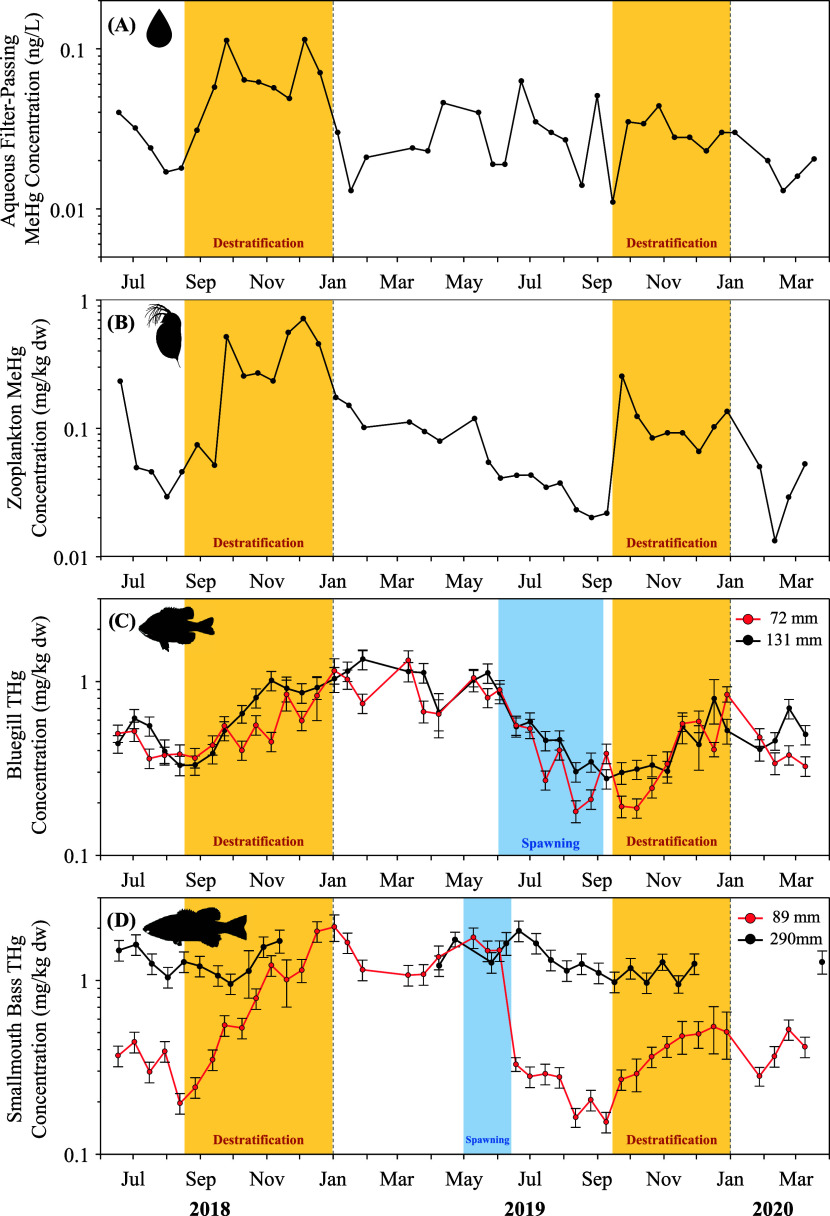
Methylmercury (MeHg) or total mercury (THg) concentrations (±standard error) in (A) filter-passing water; (B) zooplankton; (C) two sizes (72 mm and 131 mm) of Bluegill; and (D) two sizes (89 mm and 290 mm) of Smallmouth Bass from Brownlee Reservoir between June 2018 and March 2020. Yellow shading indicates the approximate destratification period for Brownlee Reservoir in each year. Blue shading indicates typical spawning period for Bluegill and Smallmouth Bass in Brownlee Reservoir.

Between June 2018 and March 2020, filter-passing MeHg varied by 11-fold, with the largest fluctuations associated with destratification, when MeHg accumulated in meta- and hypolimnions during summer stratification is mobilized throughout the water column (Figure 1A).^31,32,50^ In 2018, destratification coincided with a 6.6-fold increase in aqueous MeHg concentrations, whereas in 2019 concentrations were similarly variable before and after destratification (Figure 1A). Across both years, filter-passing MeHg concentrations differed among stratification classifications of Brownlee Reservoir (*F*_2,12.0_ = 25.2, *p* < 0.001; Table 1), with the highest concentrations observed during periods when the reservoir was destratifying (0.039 ± 0.002 ng/L; *n* = 21), intermediate concentrations during stratification (0.030 ± 0.002 ng/L; *n* = 12), and the lowest concentrations when the reservoir water column was mixed (0.019 ± 0.002 ng/L; *n* = 9; Tukey HSD *p* < 0.05).

**Table 1:** Statistical Results of Models Testing the Effect of Stratification Status on Aqueous and Biological Methylmercury (MeHg) and Total Mercury (THg) in Brownlee Reservoir, USA

**model term**		**DF**	***F* ratio**	***p*****-value**
*Aqueous MeHg*				
	stratification status	2, 10.9	33.34	<0.001
	year	1, 3.2	19.45	<0.001
	year X stratification status	2, 5.8	17.72	<0.001
*Zooplankton MeHg*				
	stratification status	2, 22.2	21.09	<0.001
	year	1, 28.2	53.57	<0.001
	year X stratification status	2, 8.3	7.86	<0.001
*Bluegill THg – juveniles*				
	stratification status	2, 0.9	2.98	0.064
	year	1, 1.1	7.24	0.011
	year X stratification status	2, 2	6.45	0.004
*Bluegill THg – adults*				
	stratification status	2, 1.3	5.57	0.008
	year	1, 1	8.58	0.006
	year X stratification status	2, 1.8	7.94	0.001
*Smallmouth Bass THg – juveniles*				
	stratification status	2, 1.4	1.89	0.166
	year	1, 1.7	4.81	0.035
	year X stratification status	2, 4.5	6.23	0.005
*Smallmouth Bass THg – adults*				
	stratification status	2, 0.2	2.98	0.072
	year	1, 0	0.00	0.949
	year X stratification status	2, 0.1	1.28	0.299

Zooplankton MeHg concentrations varied by over 53-fold over the course of sampling (Figure 1B). As with aqueous MeHg, zooplankton MeHg concentrations varied seasonally, with the highest concentrations coinciding with destratification (Figure 1B). During the initial erosion of the metalimnion in late August through September, zooplankton MeHg concentrations increased by 24-fold in 2018 and 13-fold in 2019. Overall, mean MeHg concentrations in zooplankton were higher during destratification (123.3 ± 11.7 ng/g dw) than during mixed or stratified periods (67.7 ± 10.0 and 55.3 ± 7.1 ng/g dw, respectively; *F*_2,167_ = 13.7, *p* < 0.001; Table 1); however, like aqueous MeHg, the magnitude of this increase differed between years (*F*_2,167_ = 7.9, *p* < 0.001). During the 2018 sampling year (June 2018 – March 2019), the mean MeHg concentration during destratification (220.1 ± 25.3 ng/g dw) was 3.5-fold higher than the mean MeHg concentration when stratified (62.8 ± 11.4 ng/g dw), and 1.8-fold higher than the mixed period mean MeHg concentration (123.2 ± 20.0 ng/g dw). In 2019, the highest mean MeHg concentration was also observed during destratification (70.6 ng/g dw), although this mean was not statistically different than the mean concentration during the stratified period (51.9 ± 6.7 ng/g dw; Tukey HSD *p* > 0.05). The mixed period in 2019 had the lowest mean MeHg concentration (32.0 ± 5.8 ng/g dw); approximately half the concentration observed during destratification.

We examined seasonal patterns in the THg concentrations of juvenile and adult Bluegill and Smallmouth Bass from Brownlee Reservoir. As with water and zooplankton, there was substantial variation in fish THg concentrations over the sampling period (2- to 13-fold depending on species and size-class; Figure 1C,D). Specifically, across the nearly two years of biweekly sampling, THg concentrations in adult bass varied relatively little (<2-fold) and did not differ with reservoir stratification status (Figure 1D; Table 1). In contrast, THg concentrations in adult Bluegill, juvenile Bluegill, and juvenile bass varied by 5-fold, 3-fold, and 13-fold, respectively, over the sampling period. Although seasonal patterns differed among species and size-classes (Table 1), much of the variation observed was associated with destratification, during which mean THg concentrations increased between 3-fold in Bluegill and 11-fold in juvenile bass during destratification (Figure 1C,D). Concentrations of THg in Bluegill and juvenile bass did not immediately decline following destratification, instead remaining elevated through the winter and early spring, until early summer when concentrations gradually (Bluegill) or abruptly (juvenile bass) declined (Figure 1C,D).

Our results across multiple matrices implicate the buildup of aqueous MeHg produced in the meta- and hypolimnion–and subsequent mixing through the water column during destratification–as a key process influencing seasonal patterns of MeHg within Brownlee Reservoir^15,50^ and MeHg exposure to the reservoir’s food web.^31,32,34^ The seasonal increase in zooplankton MeHg concentrations that coincided with destratification may be of particular influence because the initial incorporation of MeHg at the base of food webs often represents the largest increase in concentrations between trophic steps^63^ and can determine MeHg exposure to higher trophic positions.^64,65^ Two prior studies have reported a similar range of increases in zooplankton and planktivorous fish MeHg during destratification, with 1.3 to 4.0 times higher concentrations than during stratified or mixed sampling periods.^31,32^ Further, the accumulation of MeHg in lower trophic position biota also affects the export of MeHg through the dam to downstream habitats, particularly via plankton, which represent the largest biotic pool of exported MeHg,^14,66^ and likely contribute disproportionally to elevated MeHg concentrations in downstream fisheries.^33,34,66^

Across matrices, the 2019 change in MeHg concentrations associated with destratification was muted compared to the change in 2018. This observation coincides with approximately 25% lower peak volume of hypoxic water in 2019 compared with in 2018 (Figure S2),^14^ and 2019 having among the lowest volume of hypoxic water recorded since 1998.^51^ The extent of hypoxia is known to influence the production and accumulation of aqueous MeHg within the water column,^15,30^ with implications for MeHg bioaccumulation. For example, Herrin et al.^31^ found that the increase in biotic MeHg concentrations associated with destratification was negatively correlated with hypolimnetic DO concentrations and positively correlated with the mass of MeHg accumulated in the hypolimnion prior to turnover. Similarly, the annual volume of hypoxic water in Brownlee Reservoir is positively correlated with maximum aqueous MeHg concentrations during destratification and the export of MeHg from the reservoir.^14^ This interannual variation in the severity of hypoxia is the cumulative effect of differences in incoming flows, water temperatures, nutrient concentrations, and organic matter delivery among years.^51^ Specifically, lower flows, higher inflowing water temperatures, and higher nutrient concentrations or allochthonous organic matter result in larger meta- and hypolimnions, facilitating water column MeHg production.^15,30,50,51,53^ Interestingly, reservoirs that develop sulfidic conditions in hypoxic portions of the water column and in which MeHg production occurs predominately in sediments, may respond differently to similar hydrologic controls. Under those conditions, rapid development of hypoxia in low water years may limit MeHg production in the water column.^43,67^ Together, these data provide further compelling evidence of the complex linkages between physical and biogeochemical water column conditions, MeHg production, and MeHg bioaccumulation in reservoir food webs.^15,31,32^

### 3.2 Temporal Integration across Matrices

**Figure 2:**
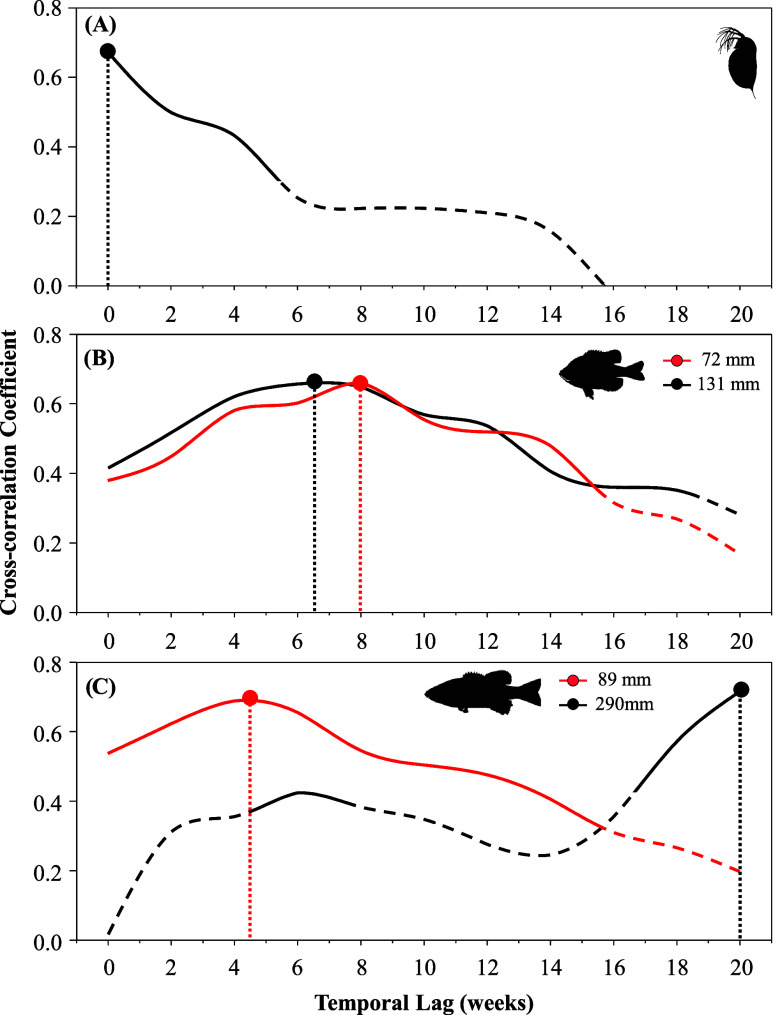
Cross-correlation functions assessing temporal lags between (A) filter-passing water methylmercury (MeHg) and zooplankton MeHg; (B) zooplankton MeHg and putative juvenile (72 mm; red line) or adult (131 mm; black line) Bluegill total mercury (THg); and (C) zooplankton MeHg and putative juvenile (89 mm; red line) or adult (290 mm; black line) Smallmouth Bass THg. Solid lines indicate statistically significant (*p* ≤ 0.05) temporal lags, dashed lines indicate nonsignificant lags. For each function, the lag with the highest correlation coefficient is indicated by the dotted lines.

**Figure 3:**
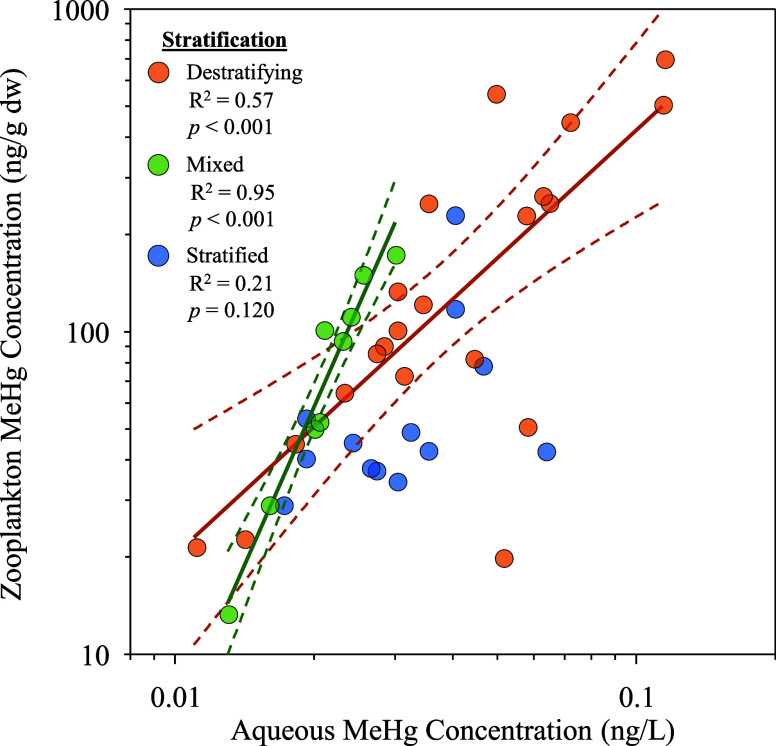
Relationships between methylmercury (MeHg) concentrations in filter-passing water and zooplankton from Brownlee Reservoir during destratifying, mixed, and stratified periods.

To better understand the rate at which MeHg mobilized from the hypoxic portion of the water column was incorporated into reservoir food webs, we tested for temporal lags between water MeHg and zooplankton MeHg, and between zooplankton MeHg and THg in each fish species and size class. Zooplankton MeHg corresponded best with water concentrations at the time of sampling, indicating that zooplankton incorporated aqueous MeHg in less than the two-week interval between sampling events (Figure 2A). Consequentially, across all sampling dates, zooplankton MeHg concentrations were positively correlated with filter-passing MeHg concentrations (*R*^2^ = 0.51, *F*_1,40_ = 40.8, *p* < 0.001; Figure S4). However, the relationship between aqueous and zooplankton MeHg concentrations varied with the stratification status of the reservoir (*F*_1,2_ = 3.6, *p* = 0.028; Figure 3). Water and zooplankton MeHg concentrations were most strongly correlated when the reservoir was fully mixed (*R*^2^ = 0.95, *F*_1,7_ = 130.0, *p* < 0.001) or destratifying (*R*^2^ = 0.57, *F*_1,19_ = 24.8, *p* < 0.001), but not when the reservoir was stratified (*R*^2^ = 0.20, *F*_1,10_ = 2.5, *p* = 0.145). The apparent decoupling between aqueous and zooplankton MeHg concentrations during stratified periods could result from our pairing epilimnetic (<10 m) zooplankton concentrations with concentrations in water flowing through the dam, which is drawn from a portion of the water column spanning approximately 25–40 m below the surface (at full pool).^53^ When the reservoir is fully stratified, there may be sufficient isolation between these strata to weaken the relationship between the two matrices. However, several additional processes could also contribute to these findings. Increased primary productivity and differences in plankton communities during the summer growing season may obscure relationships with aqueous MeHg availability and alter MeHg bioaccumulation.^65,68^ In particular, localized, episodic cyanobacteria blooms in the reservoir or upstream Snake River during the summer could moderate the net accumulation of aqueous MeHg into zooplankton since the cyanobacteria readily accumulate MeHg^63^ but are rarely consumed by most zooplankton,^69^ thus effectively sequestering the accumulated MeHg from the larger reservoir food web until the MeHg is released during decomposition.

**Figure 4:**
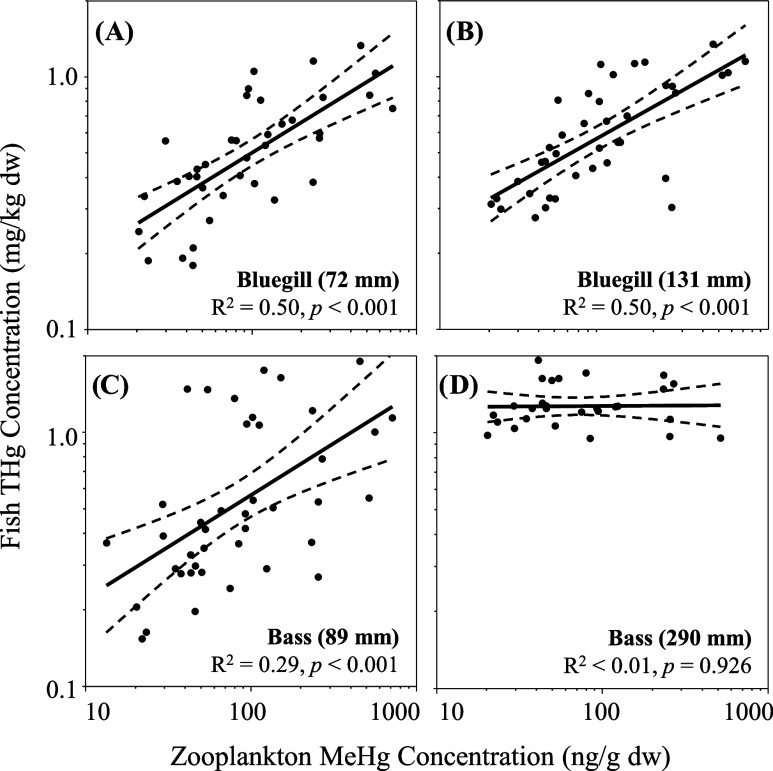
Relationships between zooplankton methylmercury (MeHg) concentrations and total mercury (THg) concentrations in (A) putative juvenile (72 mm) Bluegill; (B) adult (131 mm) Bluegill; (C) putative juvenile (89 mm) Smallmouth Bass; and (D) adult (290 mm) Smallmouth Bass from Brownlee Reservoir between June 2018 and March 2020. Relationships account for the temporal lags identified in cross-correlation analysis.

Fish THg concentrations exhibited a temporal lag in response to seasonal fluctuations in aqueous and zooplankton MeHg concentrations. In planktivorous fishes (Bluegill and juvenile bass), THg concentrations lagged water and zooplankton concentrations by between four (in juvenile bass) and eight weeks (in juvenile Bluegill; Figure 2B,C). This rapid uptake of MeHg by planktivorous fish provides further support that the pulse of MeHg associated with destratification is readily bioavailable and a potentially important source of MeHg to reservoir food webs.^31,32^ After accounting for this temporal lag, THg concentrations in Bluegill (*R*^2^ = 0.50, *p* < 0.001 for both size-classes) and juvenile bass (*R*^2^ = 0.29, *p* < 0.001) were correlated with zooplankton concentrations (Figure 4A–C). It is likely that the lower coefficient of determination between zooplankton and juvenile bass reflects ontogenetic shifts toward alternative prey (e.g., benthos or small fishes) in larger individuals.^70^ In comparison to the planktivorous fishes, piscivorous adult bass were not correlated with zooplankton concentrations (*R*^2^ < 0.01, *p* = 0.926; Figure 4D), although there was some evidence that adult bass concentrations were influenced by zooplankton concentrations over longer time frames (i.e., 20 or more weeks; Figure 2C). The lack of an immediate response in adult bass is consistent with their large body size integrating trends in MeHg availability that span the seasonal pulses associated with reservoir destratification.^71,72^ For example, populations of large-bodied fishes exposed to isotopically enriched Hg in a whole-ecosystem (e.g., a lake and surrounding watershed) experiment required more than three years to reach steady state with the addition, and eight years to fully depurate the enriched Hg following the cessation of Hg additions.^73,74^ In contrast, small, planktivorous fish fully reflected Hg from the additions within two months and started to decline within a year of additions stopping.^73,74^ However, this is not to suggest adult bass are unaffected by seasonal destratification. Total Hg concentrations in adult bass were higher in stratifying reservoirs than reservoirs that do not stratify and the greatest differences in concentrations observed in the largest size-class of bass,^34^ suggesting the effects of seasonal exposure during destratification accumulate over the lifespan of individuals. The cumulative effects of multiple destratification events may also contribute to higher THg concentrations in reservoirs with less stable stratification.^34,75^ Lakes, reservoirs, and rivers display a wide range of stratification regimes. Although some, like Brownlee Reservoir, exhibit consistently strong seasonal stratification, others have less consistent stratification patterns and may experience multiple destratification events–and associated MeHg pulses–within a year.^8,76,77^ Food webs experiencing such conditions could be regularly exposed to MeHg from these pulses, resulting in accumulation of higher MeHg concentrations, as has been observed in biota from reservoirs with inconsistent stratification regimes.^34,75^

Bluegill and juvenile bass THg concentrations remained elevated through winter and the start of summer before declining (Figure 1C,D). The causes of these declines were not specifically evaluated in this study, although three mechanisms are likely to have contributed: (1) consumption of lower Hg prey,^78^ (2) somatic growth dilution,^79^ and (3) demographic changes in the population sampled.^80,81^ The drop in Bluegill and juvenile bass THg concentrations followed several months of declining MeHg concentrations in zooplankton prey (Figure 1B), suggesting reduced dietary exposure, an important factor influencing bioaccumulation.^64^ Further, the influence of lower prey MeHg concentrations was likely compounded by increased feeding and growth rates through the spring and early summer,^82,83^ potentially exacerbating declines in fish THg via somatic growth dilution.^79^ Together, these processes likely account for the gradual declines observed in THg of both Bluegill size classes. In contrast, the substantial drop observed in THg concentrations of juvenile Smallmouth Bass appears to reflect the recruitment of a new cohort into the population that has not been exposed to a MeHg pulse yet, as the decline occurred shortly after the typical bass spawning period in Brownlee Reservoir (Figure 1D)^54^ and was accompanied by a reduction in the average size of sampled bass from 100 to 50 mm, which correspond to age-1 and age-0 Smallmouth Bass, respectively, in Brownlee Reservoir.^54,83^ Unlike Smallmouth Bass, which have a short, synchronized spawning period and distinct age cohorts, Bluegill spawn over a protracted period and young-of-year display a continuum of size that often overlap among age-classes,^84,85^ making it difficult to detect a similar cohort effect in Bluegill. Further, Bluegill reach sexual maturity at a younger age and wider range of sizes than Smallmouth Bass,^84,86^ and it is likely that some of the “juvenile” Bluegill sampled were mature individuals. This may have also contributed to the lack of an obvious cohort effect in Bluegill as well as the similarity in patterns for the two Bluegill size-classes.

### 3.3 Implications

Through high-frequency, biweekly sampling over nearly two years we demonstrate substantial seasonal variability in Hg concentrations within the reservoir that are consistent among matrices (water, zooplankton, fish). Given the timing of changes in MeHg concentrations across matrices, our findings suggest that destratification results in pulses of bioavailable MeHg from hypoxic portions of the water column that are readily incorporated into plankton and planktivorous fishes. Understanding the magnitude and timing of responses in biotic Hg concentrations to changes in MeHg exposure is an important component of developing and evaluating the effectiveness of management actions. The protracted integration of MeHg into large, piscivorous fishes complicates the assessment of trends and in many cases precludes the attribution of changes to short-term, or one-time management actions.^35,36,74^ In contrast, our findings indicate that smaller, lower trophic level species (such as planktivorous fishes) may be more effective indicators of discrete management actions because they respond more rapidly to changes in MeHg exposure and often reflect seasonal or interannual variation in the conditions influencing Hg cycling.^36,87^

The processes examined in this study are dynamic and sensitive to local, regional, and global changes in hydrologic and climatic conditions. In fact, the occurrence and duration of hypoxic conditions are increasing in lakes, reservoirs, and rivers around the globe,^7−11^ with effects on a variety of biogeochemical and ecological processes.^12^ The increasing occurrence of short-term–lasting from days to months–hypoxia in waterbodies that did not historically develop hypoxia^7,8^ may be of particular note since the more frequent breakdown in stratification could exacerbate MeHg accumulation in biota.^34,75^ MeHg bioaccumulation could also be facilitated by an increased duration or volume of hypoxia even in those waterbodies that have traditionally experienced hypoxic conditions. Understanding the changes in stratification regimes and associated impacts to MeHg production and bioaccumulation are key components to predicting and managing the broader impacts of global change on Hg cycling.^88^

Our results highlight the how the well-established linkages between nutrient and organic matter loading, physical water column structure, and biogeochemical processes in the formation of MeHg^15,30^ can dictate MeHg bioaccumulation in some aquatic food webs. These findings suggest that reducing MeHg concentrations in fish from these habitats may require a more holistic approach that addresses the associations among these processes and extends beyond an individual waterbody. For example, from 1995 to 2021, efforts to improve water quality in the Snake River decreased nutrient and chlorophyll *a* concentrations flowing into Brownlee Reservoir, leading to a 33% reduction in the volume of hypoxic water in the reservoir.^51^ Given the demonstrated importance of hypoxic conditions to water-column MeHg production in Brownlee Reservoir,^15,30^ these changes have implications for MeHg loads and transport through the Hells Canyon Complex,^14,50^ as well as, influences on MeHg concentrations in reservoir and downstream fishes.^34^ The combined results of these and the current study, highlight the value of an integrated, process-driven management strategy for the mitigation of MeHg risk to ecosystem and human health.

## Supporting Information Available

Table summarizing of quality assurance results, a study map, and three figures for supporting analyses (PDF)

## References

[ref1] Bravo A. G.; Cosio C. Biotic formation of methylmercury: A bio–physico–chemical conundrum. Limnol. Oceanogr. 2020, 65 (5), 1010–1027. 10.1002/lno.11366. 32612306 PMC7319479

[ref2] Regnell O.; Watras C. J. Microbial Mercury Methylation in Aquatic Environments: A Critical Review of Published Field and Laboratory Studies. Environ. Sci. Technol. 2019, 53 (1), 4–19. 10.1021/acs.est.8b02709. 30525497

[ref3] Dudgeon D. Multiple threats imperil freshwater biodiversity in the Anthropocene. Curr. Biol. 2019, 29 (19), R960–R967. 10.1016/j.cub.2019.08.002. 31593677

[ref4] Reid A. J.; Carlson A. K.; Creed I. F.; Eliason E. J.; Gell P. A.; Johnson P. T. J.; Kidd K. A.; MacCormack T. J.; Olden J. D.; Ormerod S. J.; Smol J. P.; Taylor W. W.; Tockner K.; Vermaire J. C.; Dudgeon D.; Cooke S. J. Emerging threats and persistent conservation challenges for freshwater biodiversity. Biol. Rev. 2019, 94 (3), 849–873. 10.1111/brv.12480. 30467930

[ref5] Woolway R. I.; Sharma S.; Smol J. P. Lakes in Hot Water: The Impacts of a Changing Climate on Aquatic Ecosystems. BioScience 2022, 72 (11), 1050–1061. 10.1093/biosci/biac052. 36325103 PMC9618276

[ref6] Wrona F. J.; Prowse T. D.; Reist J. D.; Hobbie J. E.; Lévesque L. M. J.; Vincent W. F. Climate Change Effects on Aquatic Biota, Ecosystem Structure and Function. Ambio 2006, 35 (7), 359–369. 10.1579/0044-7447(2006)35[359:cceoab]2.0.co;2. 17256640

[ref7] Blaszczak J. R.; Koenig L. E.; Mejia F. H.; Gómez-Gener L.; Dutton C. L.; Carter A. M.; Grimm N. B.; Harvey J. W.; Helton A. M.; Cohen M. J. Extent, patterns, and drivers of hypoxia in the world’s streams and rivers. Limnol. Oceanogr. Lett. 2023, 8 (3), 453–463. 10.1002/lol2.10297.

[ref8] Jane S. F.; Hansen G. J. A.; Kraemer B. M.; Leavitt P. R.; Mincer J. L.; North R. L.; Pilla R. M.; Stetler J. T.; Williamson C. E.; Woolway R. I.; Arvola L.; Chandra S.; DeGasperi C. L.; Diemer L.; Dunalska J.; Erina O.; Flaim G.; Grossart H.-P.; Hambright K. D.; Hein C.; Hejzlar J.; Janus L. L.; Jenny J.-P.; Jones J. R.; Knoll L. B.; Leoni B.; Mackay E.; Matsuzaki S.-I. S.; McBride C.; Müller-Navarra D. C.; Paterson A. M.; Pierson D.; Rogora M.; Rusak J. A.; Sadro S.; Saulnier-Talbot E.; Schmid M.; Sommaruga R.; Thiery W.; Verburg P.; Weathers K. C.; Weyhenmeyer G. A.; Yokota K.; Rose K. C. Widespread deoxygenation of temperate lakes. Nature 2021, 594 (7861), 66–70. 10.1038/s41586-021-03550-y. 34079137

[ref9] Jane S. F.; Mincer J. L.; Lau M. P.; Lewis A. S. L.; Stetler J. T.; Rose K. C. Longer duration of seasonal stratification contributes to widespread increases in lake hypoxia and anoxia. Global Change Biol. 2023, 29 (4), 1009–1023. 10.1111/gcb.16525. 36472079

[ref10] Jansen J.; Simpson G. L.; Weyhenmeyer G. A.; Härkönen L. H.; Paterson A. M.; del Giorgio P. A.; Prairie Y. T. Climate-driven deoxygenation of northern lakes. Nature Climate Change 2024, 14, 832 10.1038/s41558-024-02058-3.

[ref11] Zhi W.; Klingler C.; Liu J.; Li L. Widespread deoxygenation in warming rivers. Nature Climate Change 2023, 13 (10), 1105–1113. 10.1038/s41558-023-01793-3.

[ref12] LaBrie R.; Hupfer M.; Lau M. P. Anaerobic duration predicts biogeochemical consequences of oxygen depletion in lakes. Limnology and Oceanography Letters 2023, 8 (4), 666–674. 10.1002/lol2.10324.

[ref13] Sunderland E. M.; Krabbenhoft D. P.; Moreau J. W.; Strode S. A.; Landing W. M. Mercury sources, distribution, and bioavailability in the North Pacific Ocean: Insights from data and models. Global Biogeochem. Cycles 2009, 23 (2), GB2010 10.1029/2008GB003425.

[ref14] Baldwin A. K.; Eagles-Smith C. A.; Willacker J. J.; Poulin B. A.; Krabbenhoft D. P.; Naymik J.; Tate M. T.; Bates D.; Gastelecutto N.; Hoovestol C.; Larsen C.; Yoder A. M.; Chandler J.; Myers R. In-Reservoir Physical Processes Modulate Aqueous and Biological Methylmercury Export from a Seasonally Anoxic Reservoir. Environ. Sci. Technol. 2022, 56 (19), 13751–13760. 10.1021/acs.est.2c03958. 36107858 PMC9535939

[ref15] Poulin B. A.; Tate M. T.; Ogorek J.; Breitmeyer S. E.; Baldwin A. K.; Yoder A. M.; Harris R.; Naymik J.; Gastelecutto N.; Hoovestol C.; Larsen C.; Myers R.; Aiken G. R.; Krabbenhoft D. P. Biogeochemical and hydrologic synergy control mercury fate in an arid land river-reservoir system. Environ. Sci.:Processes Impacts 2023, 25, 912–928. 10.1039/D3EM00032J. 37186129

[ref16] Wetzel R. G. Limnology; Academic Press, 2001.

[ref17] Bush T.; Diao M.; Allen R. J.; Sinnige R.; Muyzer G.; Huisman J. Oxic-anoxic regime shifts mediated by feedbacks between biogeochemical processes and microbial community dynamics. Nat. Commun. 2017, 8 (1), 789 10.1038/s41467-017-00912-x. 28986518 PMC5630580

[ref18] O’Reilly C. M.; Sharma S.; Gray D. K.; Hampton S. E.; Read J. S.; Rowley R. J.; Schneider P.; Lenters J. D.; McIntyre P. B.; Kraemer B. M.; Weyhenmeyer G. A.; Straile D.; Dong B.; Adrian R.; Allan M. G.; Anneville O.; Arvola L.; Austin J.; Bailey J. L.; Baron J. S.; Brookes J. D.; de Eyto E.; Dokulil M. T.; Hamilton D. P.; Havens K.; Hetherington A. L.; Higgins S. N.; Hook S.; Izmest’eva L. R.; Joehnk K. D.; Kangur K.; Kasprzak P.; Kumagai M.; Kuusisto E.; Leshkevich G.; Livingstone D. M.; MacIntyre S.; May L.; Melack J. M.; Mueller-Navarra D. C.; Naumenko M.; Noges P.; Noges T.; North R. P.; Plisnier P.-D.; Rigosi A.; Rimmer A.; Rogora M.; Rudstam L. G.; Rusak J. A.; Salmaso N.; Samal N. R.; Schindler D. E.; Schladow S. G.; Schmid M.; Schmidt S. R.; Silow E.; Soylu M. E.; Teubner K.; Verburg P.; Voutilainen A.; Watkinson A.; Williamson C. E.; Zhang G. Rapid and highly variable warming of lake surface waters around the globe. Geophys. Res. Lett. 2015, 42 (24), 10,773–710,781. 10.1002/2015GL066235.

[ref19] Smith V. H.; Tilman G. D.; Nekola J. C. Eutrophication: impacts of excess nutrient inputs on freshwater, marine, and terrestrial ecosystems. Environ. Pollut. 1999, 100 (1), 179–196. 10.1016/S0269-7491(99)00091-3. 15093117

[ref20] Murphy G. E. P.; Romanuk T. N.; Worm B. Cascading effects of climate change on plankton community structure. Ecol. Evol. 2020, 10 (4), 2170–2181. 10.1002/ece3.6055. 32128147 PMC7042755

[ref21] Chapra S. C.; Boehlert B.; Fant C.; Bierman V. J.; Henderson J.; Mills D.; Mas D. M. L.; Rennels L.; Jantarasami L.; Martinich J.; Strzepek K. M.; Paerl H. W. Climate Change Impacts on Harmful Algal Blooms in U.S. Freshwaters: A Screening-Level Assessment. Environ. Sci. Technol. 2017, 51, 8933 10.1021/acs.est.7b01498. 28650153

[ref22] Wu Z.; Yu D.; Yu Q.; Liu Q.; Zhang M.; Dahlgren R. A.; Middelburg J. J.; Qu L.; Li Q.; Guo W.; Chen N. Greenhouse gas emissions (CO2–CH4–N2O) along a large reservoir-downstream river continuum: The role of seasonal hypoxia. Limnol. Oceanogr. 2024, 69 (5), 1015–1029. 10.1002/lno.12544.

[ref23] Carey C. C.; Hanson P. C.; Thomas R. Q.; Gerling A. B.; Hounshell A. G.; Lewis A. S. L.; Lofton M. E.; McClure R. P.; Wander H. L.; Woelmer W. M.; Niederlehner B. R.; Schreiber M. E. Anoxia decreases the magnitude of the carbon, nitrogen, and phosphorus sink in freshwaters. Global Change Biol. 2022, 28 (16), 4861–4881. 10.1111/gcb.16228. PMC9543840 35611634

[ref24] Tye S. P.; Siepielski A. M.; Bray A.; Rypel A. L.; Phelps N. B. D.; Fey S. B. Climate warming amplifies the frequency of fish mass mortality events across north temperate lakes. Limnol. Oceanogr. Lett. 2022, 7 (6), 510–519. 10.1002/lol2.10274.

[ref25] Sundseth K.; Pacyna J. M.; Pacyna E. G.; Pirrone N.; Thorne R. J. Global Sources and Pathways of Mercury in the Context of Human Health. Int. J. Environ. Res. Public Health 2017, 14 (1), 105 10.3390/ijerph14010105. 28117743 PMC5295355

[ref26] Cleary B. M.; Romano M. E.; Chen C. Y.; Heiger-Bernays W.; Crawford K. A. Comparison of Recreational Fish Consumption Advisories Across the USA. Curr. Environ. Health Rep. 2021, 8 (2), 71–88. 10.1007/s40572-021-00312-w. 33934293 PMC8208921

[ref27] Evers D. C.; Ackerman J. T.; Åkerblom S.; Bally D.; Basu N.; Bishop K.; Bodin N.; Braaten H. F. V.; Burton M. E. H.; Bustamante P.; Chen C.; Chételat J.; Christian L.; Dietz R.; Drevnick P.; Eagles-Smith C.; Fernandez L. E.; Hammerschlag N.; Harmelin-Vivien M.; Harte A.; Krümmel E. M.; Brito J. L.; Medina G.; Barrios Rodriguez C. A.; Stenhouse I.; Sunderland E.; Takeuchi A.; Tear T.; Vega C.; Wilson S.; Wu P. Global mercury concentrations in biota: their use as a basis for a global biomonitoring framework. Ecotoxicology 2024, 33 (4), 325–396. 10.1007/s10646-024-02747-x. 38683471 PMC11213816

[ref28] Pacyna J. M.; Travnikov O.; De Simone F.; Hedgecock I. M.; Sundseth K.; Pacyna E. G.; Steenhuisen F.; Pirrone N.; Munthe J.; Kindbom K. Current and future levels of mercury atmospheric pollution on a global scale. Atmos. Chem. Phys. 2016, 16 (19), 12495–12511. 10.5194/acp-16-12495-2016.

[ref29] Eckley C. S.; Hintelmann H. Determination of mercury methylation potentials in the water column of lakes across Canada. Sci. Total Environ. 2006, 368 (1), 111–125. 10.1016/j.scitotenv.2005.09.042. 16216310

[ref30] Peterson B. D.; Poulin B. A.; Krabbenhoft D. P.; Tate M. T.; Baldwin A. K.; Naymik J.; Gastelecutto N.; McMahon K. D. Metabolically diverse microorganisms mediate methylmercury formation under nitrate-reducing conditions in a dynamic hydroelectric reservoir. ISME J. 2023, 17 (10), 1705–1718. 10.1038/s41396-023-01482-1. 37495676 PMC10504345

[ref31] Herrin R. T.; Lathrop R. C.; Gorski P. R.; Andren A. W. Hypolimnetic methylmercury and its uptake by plankton during fall destratification: A key entry point of mercury into lake food chains?. Limnol. Oceanogr. 1998, 43 (7), 1476–1486. 10.4319/lo.1998.43.7.1476.

[ref32] Slotton D. G.; Reuter J. E.; Goldman C. R. Mercury uptake patterns of biota in a seasonally anoxic northern California Reservoir. Water, Air, Soil Pollut. 1995, 80 (1–4), 841–850. 10.1007/BF01189735.

[ref33] Kasper D.; Forsberg B. R.; Amaral J. H. F.; Leitão R. P.; Py-Daniel S. S.; Bastos W. R.; Malm O. Reservoir Stratification Affects Methylmercury Levels in River Water, Plankton, and Fish Downstream from Balbina Hydroelectric Dam, Amazonas, Brazil. Environ. Sci. Technol. 2014, 48 (2), 1032–1040. 10.1021/es4042644. 24397364

[ref34] Willacker J. J.; Eagles-Smith C. A.; Chandler J. A.; Naymik J.; Myers R.; Krabbenhoft D. P. Reservoir Stratification Modulates the Influence of Impoundments on Fish Mercury Concentrations along an Arid Land River System. Environ. Sci. Technol. 2023, 57 (50), 21313–21326. 10.1021/acs.est.3c04646. 38051342 PMC10734268

[ref35] Wang F.; Outridge P. M.; Feng X.; Meng B.; Heimbürger-Boavida L.-E.; Mason R. P. How closely do mercury trends in fish and other aquatic wildlife track those in the atmosphere? – Implications for evaluating the effectiveness of the Minamata Convention. Sci. Total Environ. 2019, 674, 58–70. 10.1016/j.scitotenv.2019.04.101. 31003088

[ref36] Wiener J.; Bodaly R.; Brown S.; Lucotte M.; Newman M.; Porcella D.; Reash R.; Swain E. Monitoring and evaluating trends in methylmercury accumulation in aquatic biota. Ecosyst. Responses Mercury Contam. 2007, 87–122. 10.1201/9780849388897.ch4.

[ref37] Friedl G.; Wüest A. Disrupting biogeochemical cycles-Consequences of damming. Aquat. Sci. 2002, 64 (1), 55–65. 10.1007/s00027-002-8054-0.

[ref38] Willacker J. J.; Eagles-Smith C. A.; Lutz M. A.; Tate M. T.; Lepak J. M.; Ackerman J. T. Reservoirs and water management influence fish mercury concentrations in the western United States and Canada. Sci. Total Environ. 2016, 568 (2016), 739–748. 10.1016/j.scitotenv.2016.03.050. 27039275

[ref39] Hall B.; Louis V. S.; Rolfhus K.; Bodaly R.; Beaty K.; Paterson M.; Cherewyk K. P. Impacts of reservoir creation on the biogeochemical cycling of methyl mercury and total mercury in boreal upland forests. Ecosystems 2005, 8 (3), 248–266. 10.1007/s10021-003-0094-3.

[ref40] Eckley C. S.; Luxton T. P.; Goetz J.; McKernan J. Water-level fluctuations influence sediment porewater chemistry and methylmercury production in a flood-control reservoir. Environ. Pollut. 2017, 222, 32–41. 10.1016/j.envpol.2017.01.010. 28104341 PMC6498431

[ref41] Li G.; Wang X. T.; Yang Z.; Mao C.; West A. J.; Ji J. Dam-triggered organic carbon sequestration makes the Changjiang (Yangtze) river basin (China) a significant carbon sink. J. Geophys. Res.:Biogeosci. 2015, 120 (1), 39–53. 10.1002/2014JG002646.

[ref42] Maavara T.; Lauerwald R.; Regnier P.; Van Cappellen P. Global perturbation of organic carbon cycling by river damming. Nat. Commun. 2017, 8 (1), 15347 10.1038/ncomms15347. 28513580 PMC5442313

[ref43] Millard G.; Eckley C. S.; Luxton T. P.; Krabbenhoft D.; Goetz J.; McKernan J.; DeWild J. Evaluating the influence of seasonal stratification on mercury methylation rates in the water column and sediment in a contaminated section of a western U.S.A. reservoir. Environ. Pollut. 2023, 316, 120485 10.1016/j.envpol.2022.120485. 36279994 PMC10259237

[ref44] Lehner B.; Reidy Liermann C.; Revenga C.; Vorosmarty C.; Fekete B.; Crouzet P.; Doll P.; Endejan M.; Frenken K.; Magome J.; Nilsson C.; Robertson J. C.; Rodel R.; Sindorf N.; Wisser D. High-Resolution Mapping of the World’s Reservoirs and Dams for Sustainable River-Flow Management. Front. Ecol. Environ. 2011, 9, 494–502. 10.1890/100125.

[ref45] Nilsson C.; Reidy C. A.; Dynesius M.; Revenga C. Fragmentation and Flow Regulation of the World’s Large River Systems. Science 2005, 308 (5720), 405–408. 10.1126/science.1107887. 15831757

[ref46] Graf W. L. Dam nation: A geographic census of American dams and their large-scale hydrologic impacts. Water Resour. Res. 1999, 35 (4), 1305–1311. 10.1029/1999WR900016.

[ref47] Parisek C. A.; De Castro F. A.; Colby J. D.; Leidy G. R.; Sadro S.; Rypel A. L. Reservoir ecosystems support large pools of fish biomass. Sci. Rep. 2024, 14 (1), 9428 10.1038/s41598-024-59730-z. 38658610 PMC11043325

[ref48] Hutt C. P.; Hunt K. M.; Steffen S. F.; Grado S. C.; Miranda L. E. Economic Values and Regional Economic Impacts of Recreational Fisheries in Mississippi Reservoirs. North Am. J. Fish. Manage. 2013, 33 (1), 44–55. 10.1080/02755947.2012.739986.

[ref49] Melstrom R. T.; Kaemingk M. A.; Cole N. W.; Whitehead J. C.; Chizinski C. J.; Pope K. L. Valuing Angling on Reservoirs Using Benefit Transfer. North Am. J. Fish. Manage. 2023, 43 (2), 400–416. 10.1002/nafm.10802.

[ref50] Baldwin A. K.; Poulin B. A.; Naymik J.; Hoovestol C.; Clark G. M.; Krabbenhoft D. P. Seasonal Dynamics and Interannual Variability in Mercury Concentrations and Loads through a Three-Reservoir Complex. Environ. Sci. Technol. 2020, 54 (15), 9305–9314. 10.1021/acs.est.9b07103. 32667810

[ref51] Naymik J.; Larsen C. A.; Myers R.; Hoovestol C.; Gastelecutto N.; Bates D. Long-term trends in inflowing chlorophyll a and nutrients and their relation to dissolved oxygen in a large western reservoir. Lake Reservoir Manage. 2023, 39 (1), 53–71. 10.1080/10402381.2022.2160395.

[ref52] Baldwin A. K.; Janssen S. E.; Tate M. T.; Poulin B. A.; Yoder A. M.; Naymik J.; Larsen C.; Hoovestol C.; Krabbenhoft D. P. Mercury sources and budget for the Snake River above a hydroelectric reservoir complex. Sci. Total Environ. 2024, 907, 167961 10.1016/j.scitotenv.2023.167961. 37865255

[ref53] Myers R.; Harrison J.; Parkinson S. K.; Hoelscher B.; Naymik J.; Parkinson S. E. Pollutant Transport and Processing in the Hells Canyon Complex, Technical appendices for new license application: Hells Canyon Hydroelectric Project. Technical Report E, 2001, 2.2-1.

[ref54] Richter T. Hells Canyon Complex Resident Fish Study; Technical Report Appendix E.3.1–5; 2003.

[ref55] U.S. Environmental Protection Agency. Methyl Mercury in Water by Distillation, Aqueous Ethylation, Purge and Trap, and Cold-vapor Atomic Fluorescence Spectrometry. Method 1630. EPA-821-R-01–020; Office of Water and Office of Science and Technology, Washington, DC, 2001.

[ref56] De Wild J. F.; Olsen M. L.; Olund S. D. Determination of Methyl Mercury by Aqueous Phase Ethylation, Followed by Gas Chromatographic Separation with Cold Vapor Atomic Fluorescence Detection; US Geological Survey, 2002.

[ref57] Antweiler R. C. Evaluation of Statistical Treatments of Left-Censored Environmental Data Using Coincident Uncensored Data Sets. II. Group Comparisons. Environ. Sci. Technol. 2015, 49 (22), 13439–13446. 10.1021/acs.est.5b02385. 26490190

[ref58] Helsel D. R. Fabricating data: How substituting values for nondetects can ruin results, and what can be done about it. Chemosphere 2006, 65 (11), 2434–2439. 10.1016/j.chemosphere.2006.04.051. 16737727

[ref59] JMP Software. Version 12.0.1; Cary, NC, USA, 2016.

[ref60] Williams B. K.; Nichols J. D.; Conroy M. J. Analysis and Management of Animal Populations; Academic Press, 2002.

[ref61] Eagles-Smith C. A.; Ackerman J. T.; Willacker J. J.; Tate M. T.; Lutz M. A.; Fleck J. A.; Stewart A. R.; Wiener J. G.; Evers D. C.; Lepak J. M.; Davis J. A.; Pritz C. F. Spatial and temporal patterns of mercury concentrations in freshwater fish across the Western United States and Canada. Sci. Total Environ. 2016, 568 (2016), 1171–1184. 10.1016/j.scitotenv.2016.03.229. 27102274

[ref62] Chatfield C. The Analysis of Time Series: An Introduction; Chapman and Hall/CRC, 2003 10.4324/9780203491683.

[ref63] Pickhardt P. C.; Fisher N. S. Accumulation of inorganic and methylmercury by freshwater phytoplankton in two contrasting water bodies. Environ. Sci. Technol. 2007, 41 (1), 125–131. 10.1021/es060966w. 17265937

[ref64] Chételat J.; Ackerman J. T.; Eagles-Smith C. A.; Hebert C. E. Methylmercury exposure in wildlife: A review of the ecological and physiological processes affecting contaminant concentrations and their interpretation. Sci. Total Environ. 2020, 711, 135117 10.1016/j.scitotenv.2019.135117. 31831233

[ref65] Stewart A. R.; Saiki M. K.; Kuwabara J. S.; Alpers C. N.; Marvin-DiPasquale M.; Krabbenhoft D. P. Influence of plankton mercury dynamics and trophic pathways on mercury concentrations of top predator fish of a mining-impacted reservoir. Can. J. Fish. Aquat. Sci. 2008, 65 (11), 2351–2366. 10.1139/F08-140.

[ref66] Schetagne R.; Doyon J.-F.; Fournier J.-J. Export of mercury downstream from reservoirs. Sci. Total Environ. 2000, 260 (2000), 135–145. 10.1016/S0048-9697(00)00557-X. 11032122

[ref67] Rodal-Morales N. D.; Beutel M.; Fuhrmann B.; Defeo S.; Hansen A. M.; Harmon T.; Brower S.; Pasek J. Hydrology and oxygen addition drive nutrients, metals, and methylmercury cycling in a hypereutrophic water supply reservoir. Front. Water 2024, 6, 1356994 10.3389/frwa.2024.1356994.

[ref68] Chen C. Y.; Folt C. L. High plankton densities reduce mercury biomagnification. Environ. Sci. Technol. 2005, 39 (1), 115–121. 10.1021/es0403007. 15667084

[ref69] Ger K. A.; Hansson L.-A.; Lürling M. Understanding cyanobacteria-zooplankton interactions in a more eutrophic world. Freshwater Biol. 2014, 59 (9), 1783–1798. 10.1111/fwb.12393.

[ref70] Kraemer L. D.; Evans D.; Dillon P. J. The impacts of ontogenetic dietary shifts in yellow perch (Perca flavescens) on Zn and Hg accumulation. Ecotoxicol. Environ. Saf. 2012, 78, 246–252. 10.1016/j.ecoenv.2011.11.033. 22177481

[ref71] Trudel M.; Rasmussen J. B. Predicting mercury concentration in fish using mass balance models. Ecol. Appl. 2001, 11 (2), 517–529. 10.1890/1051-0761(2001)011[0517:PMCIFU]2.0.CO;2.

[ref72] Trudel M.; Rasmussen J. B. Bioenergetics and mercury dynamics in fish: a modelling perspective. Can. J. Fish. Aquat. Sci. 2006, 63 (8), 1890–1902. 10.1139/f06-081.

[ref73] Harris R. C.; Rudd J. W. M.; Amyot M.; Babiarz C. L.; Beaty K. G.; Blanchfield P. J.; Bodaly R. A.; Branfireun B. A.; Gilmour C. C.; Graydon J. A.; Heyes A.; Hintelmann H.; Hurley J. P.; Kelly C. A.; Krabbenhoft D. P.; Lindberg S. E.; Mason R. P.; Paterson M. J.; Podemski C. L.; Robinson A.; Sandilands K. A.; Southworth G. R.; St Louis V. L.; Tate M. T. Whole-ecosystem study shows rapid fish-mercury response to changes in mercury deposition. Proc. Natl. Acad. Sci. U.S.A. 2007, 104 (42), 16586–16591. 10.1073/pnas.0704186104. 17901207 PMC2034227

[ref74] Blanchfield P. J.; Rudd J. W. M.; Hrenchuk L. E.; Amyot M.; Babiarz C. L.; Beaty K. G.; Bodaly R. A. D.; Branfireun B. A.; Gilmour C. C.; Graydon J. A.; Hall B. D.; Harris R. C.; Heyes A.; Hintelmann H.; Hurley J. P.; Kelly C. A.; Krabbenhoft D. P.; Lindberg S. E.; Mason R. P.; Paterson M. J.; Podemski C. L.; Sandilands K. A.; Southworth G. R.; St Louis V. L.; Tate L. S.; Tate M. T. Experimental evidence for recovery of mercury-contaminated fish populations. Nature 2022, 601 (7891), 74–78. 10.1038/s41586-021-04222-7. 34912113 PMC8732272

[ref75] Bravo A. G.; Cosio C.; Amouroux D.; Zopfi J.; Chevalley P.-A.; Spangenberg J. E.; Ungureanu V.-G.; Dominik J. Extremely elevated methyl mercury levels in water, sediment and organisms in a Romanian reservoir affected by release of mercury from a chlor-alkali plant. Water Res. 2014, 49, 391–405. 10.1016/j.watres.2013.10.024. 24216231

[ref76] Wagner N. D.; Osburn F. S.; Robbins C. J.; Ernst M. R.; Owens J.; Powers S. M.; Scott J. T. Lake stability and anoxia dynamics revealed from high frequency vertical profiling in a eutrophic polymictic reservoir. Inland Waters 2023, 13, 167–181. 10.1080/20442041.2022.2161264.

[ref77] Bouffard D.; Ackerman J. D.; Boegman L. Factors affecting the development and dynamics of hypoxia in a large shallow stratified lake: Hourly to seasonal patterns. Water Resour. Res. 2013, 49 (5), 2380–2394. 10.1002/wrcr.20241.

[ref78] Ward D. M.; Nislow K. H.; Folt C. L. Do low-mercury terrestrial resources subsidize low-mercury growth of stream fish? Differences between species along a productivity gradient. PLoS One 2012, 7 (11), e49582 10.1371/journal.pone.0049582. 23166717 PMC3500304

[ref79] Ward D. M.; Nislow K. H.; Chen C. Y.; Folt C. L. Rapid, efficient growth reduces mercury concentrations in stream-dwelling Atlantic salmon. Trans. Am. Fish. Soc. 2010, 139 (1), 1–10. 10.1577/T09-032.1. 20436784 PMC2861578

[ref80] Carr M. K.; Jardine T. D.; Doig L. E.; Jones P. D.; Bharadwaj L.; Tendler B.; Chételat J.; Cott P.; Lindenschmidt K.-E. Stable sulfur isotopes identify habitat-specific foraging and mercury exposure in a highly mobile fish community. Sci. Total Environ. 2017, 586, 338–346. 10.1016/j.scitotenv.2017.02.013. 28190573

[ref81] Swanson H.; Gantner N.; Kidd K. A.; Muir D. C. G.; Reist J. D. Comparison of mercury concentraions in landlocked, resident and sea-run fish (*Salvelinus Spp*.) from Nunavut, Canada. Environ. Toxicol. Chem. 2011, 30 (6), 1459–1467. 10.1002/etc.517. 21381088

[ref82] Dunsmoor L.; Bennett D.; Chandler J. Prey selectivity and growth of a planktivorous population of smallmouth bass in an Idaho reservoir. In The First International Smallmouth Bass Symposium; Southern Division American Fisheries Society: Bethesda, MD, 1991; pp 14–23.

[ref83] Rohrer R. Lake and Reservoir Investigations: Brownlee Reservoir Fish Population Dynamics, Community Structure and the Fishery. Job Performance Report, Project F-73-R-6. Idaho Department of Fish and Game: Boise, ID, 1984.

[ref84] Devries D. R.; Garvey J.; Wright R. Early life history and recruitment. In Centrarchid Fishes: Diversity, Biology, and Conservation; Cooke S. J.; Philipp D. P., Eds.; 2009; pp 105–133.

[ref85] Garvey J. E.; Herra T. P.; Leggett W. C. Protracted Reproduction in Sunfish: The Temporal Dimension in Fish Recruitment Revisited. Ecol. Appl. 2002, 12 (1), 194–205. 10.1890/1051-0761(2002)012[0194:PRISTT]2.0.CO;2.

[ref86] Cargnelli L. M.; Gross M. R. The temporal dimension in fish recruitment: birth date, body size, and size-dependent survival in a sunfish (bluegill: *Lepomis macrochirus*). Can. J. Fish. Aquat. Sci. 1996, 53 (2), 360–367. 10.1139/f95-193.

[ref87] Cerveny D.; Turek J.; Grabic R.; Golovko O.; Koba O.; Fedorova G.; Grabicova K.; Zlabek V.; Randak T. Young-of-the-year fish as a prospective bioindicator for aquatic environmental contamination monitoring. Water Res. 2016, 103, 334–342. 10.1016/j.watres.2016.07.046. 27486042

[ref88] Eagles-Smith C. A.; Silbergeld E. K.; Basu N.; Bustamante P.; Diaz-Barriga F.; Hopkins W. A.; Kidd K. A.; Nyland J. F. Modulators of mercury risk to wildlife and humans in the context of rapid global change. Ambio 2018, 47 (2), 170–197. 10.1007/s13280-017-1011-x. 29388128 PMC5794686

[ref89] Willacker J. J.; Chandler J. A.; Naymik J.; Myers R.; Krabbenhoft D. P.; Eagles-Smith C. A. Mercury in Smallmouth Bass from the Snake River; U.S. Geological Survey Data Release: USA, 2023; pp 2013–2022.

[ref90] Eagles-Smith C.; Willacker J. J.; Johnson B.; Pierce J.; Tennant L.; Rumrill C.; Herring G.; Bates D.; Bean B.; Chandler J.; Cooper C.; Doten K.; Eachus B.; Emery C.; Glidden T.; Johnson E.; Naymick J.; Randolph J.; Richter T.; Rutledge E.; Stephensen M.; Wisotzky C.; Woolen A. Mercury in biota from the Hells Canyon Complex on the Snake River; U.S. Geological Survey Data Release: Idaho and Oregon, 2025.

