# Supplementary material for: Contrasting Magnitude and Timing of Pulsed Aqueous Methylmercury Bioaccumulation across a Reservoir Food Web
Source: Environ Sci Technol. 2025 Feb 17;59(8):3884–94. doi: 10.1021/acs.est.4c10719 (PMC11883814; doi:10.1021/acs.est.4c10719)
Supplement: Supplementary file 1 — es4c10719_si_001.pdf [file es4c10719_si_001.pdf]

Supporting Information

Contrasting magnitude and timing of pulsed aqueous methylmercury bioaccumulation across a reservoir food web

James J. Willacker[1], Collin A. Eagles-Smith[1*], Austin K. Baldwin[2], Michael T. Tate[3], Brett A Poulin[4], Jesse Naymik[5], David P. Krabbenhoft[3], Ralph Myers[5], and James A. Chandler[5]

[1] U.S. Geological Survey, Forest and Rangeland Ecosystem Science Center, 3200 SW Jefferson Way, Corvallis, OR 97331, USA

[2] U.S. Geological Survey, Idaho Water Science Center, Boise, ID 83702, USA

[3] U.S. Geological Survey, Upper Midwest Water Science Center, 8505 Research Way, Middleton, WI 53562, USA

[4] Department of Environmental Toxicology, University of California at Davis, Davis, CA 95616, USA

[5] Idaho Power Company, 1221 West Idaho Street, Boise, ID 83702, USA

* Corresponding author: email: ceagles-smith@usgs.gov; phone: 541-750-0949

Any use of trade, firm, or product names is for descriptive purposes only and does not imply endorsement by the U.S. Government.

Supporting information includes one table and four figures in 6 pages

Table S1. Quality assurance-quality control (QAQC) results for analysis of methylmercury and total mercury in biological materials via U.S. Environmental Protection Agency methods 1630 and 7473, respectively.

QAQC Type	QAQC Metric	n	Mean	Standard Error
**Methylmercury**				
Certified Reference Material[1]	percent recovery			
Duplicate	relative percent difference			
Liquid Calibration Standard	percent recovery			
Matrix Spike[2]	percent recovery			
Reagent Blank	pg MeHg			
Method Detection Limit	ng/g			
Level of Quantification	ng/g			
**Total Mercury**				
Certified Reference Material[3]	percent recovery	78	97.3	0.78
Duplicate	relative percent difference	73	1.3	0.19
Liquid Calibration Standard	percent recovery	93	95.8	1.00
Reagent Blank	ng Hg	63	0.012	0.002
Method Detection Limit	mg/kg	723	0.002	0.0001
Level of Quantification	mg/kg	723	0.008	0.0001

[1] IAEA-452, IAEA-407; [2] digest spiked with MeHgCl; [3] DORM-4, TORT-3

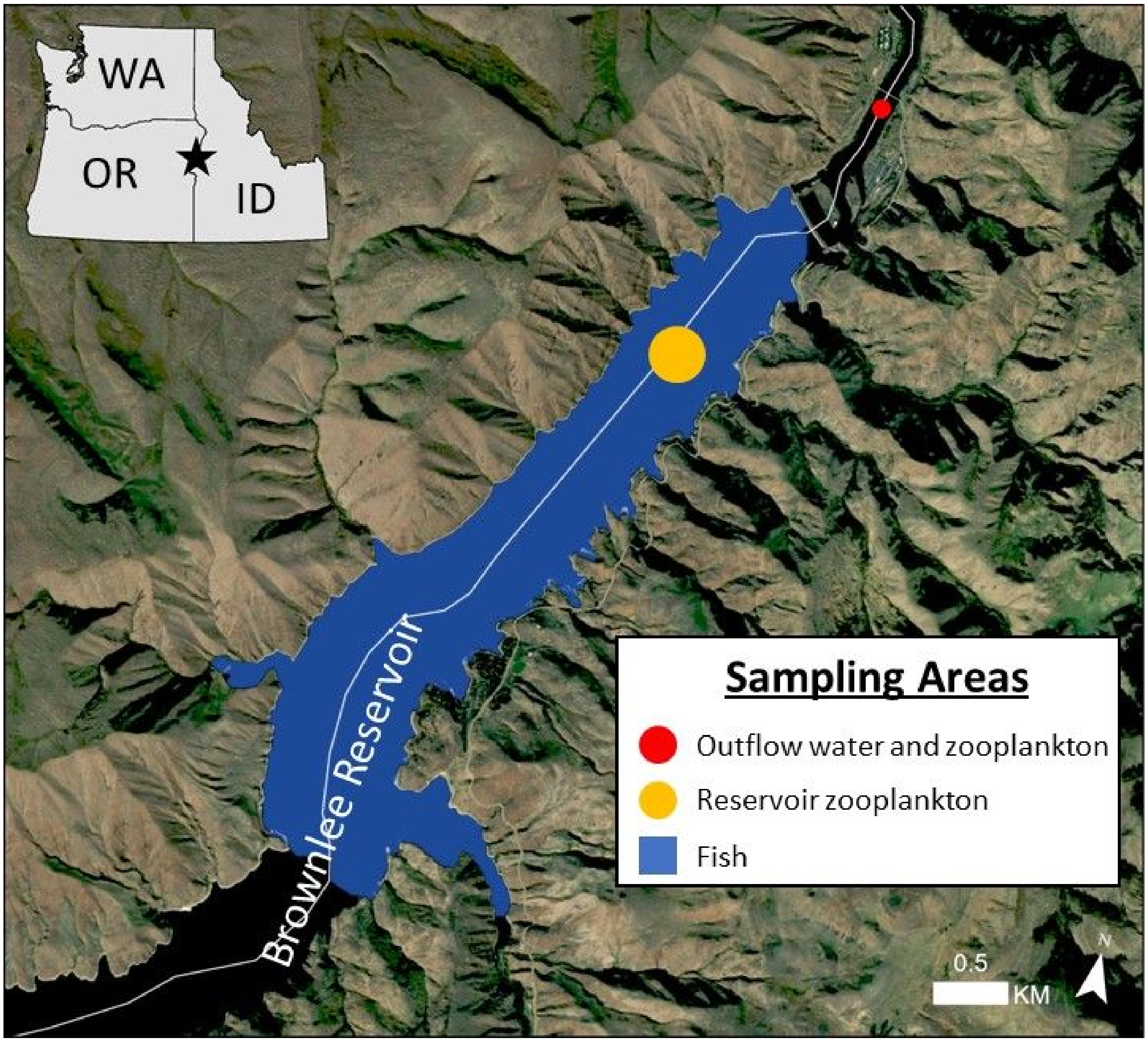

Figure S1: Map of lower Brownlee Reservoir showing areas sampled for water, zooplankton, and fish. Base map from Esri and its licensors, copyright 2024.

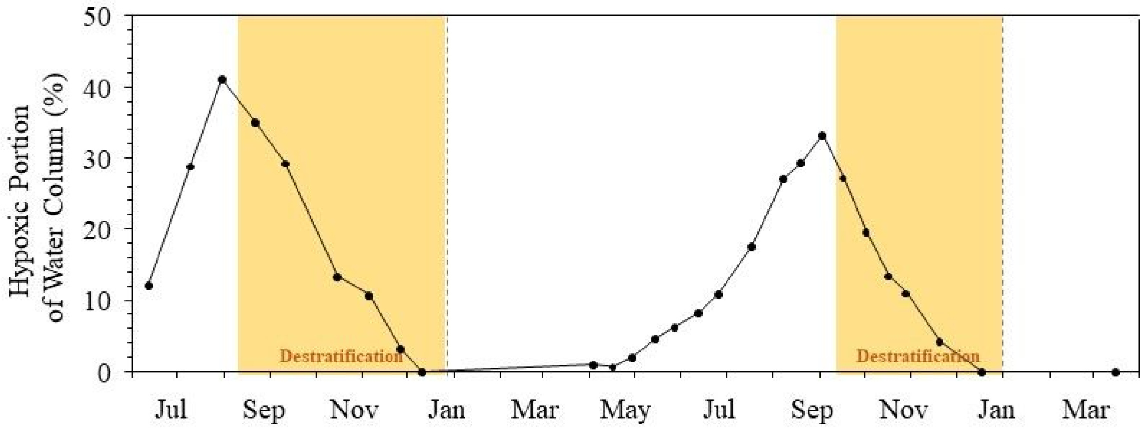

Figure S2: Timeseries showing the percent of Brownlee Reservoir water that was hypoxic (dissolved oxygen concentration < 2.0 mg/L) between June 2018 and March 2020. Yellow shading indicates the approximate destratification period for Brownlee Reservoir in each year.

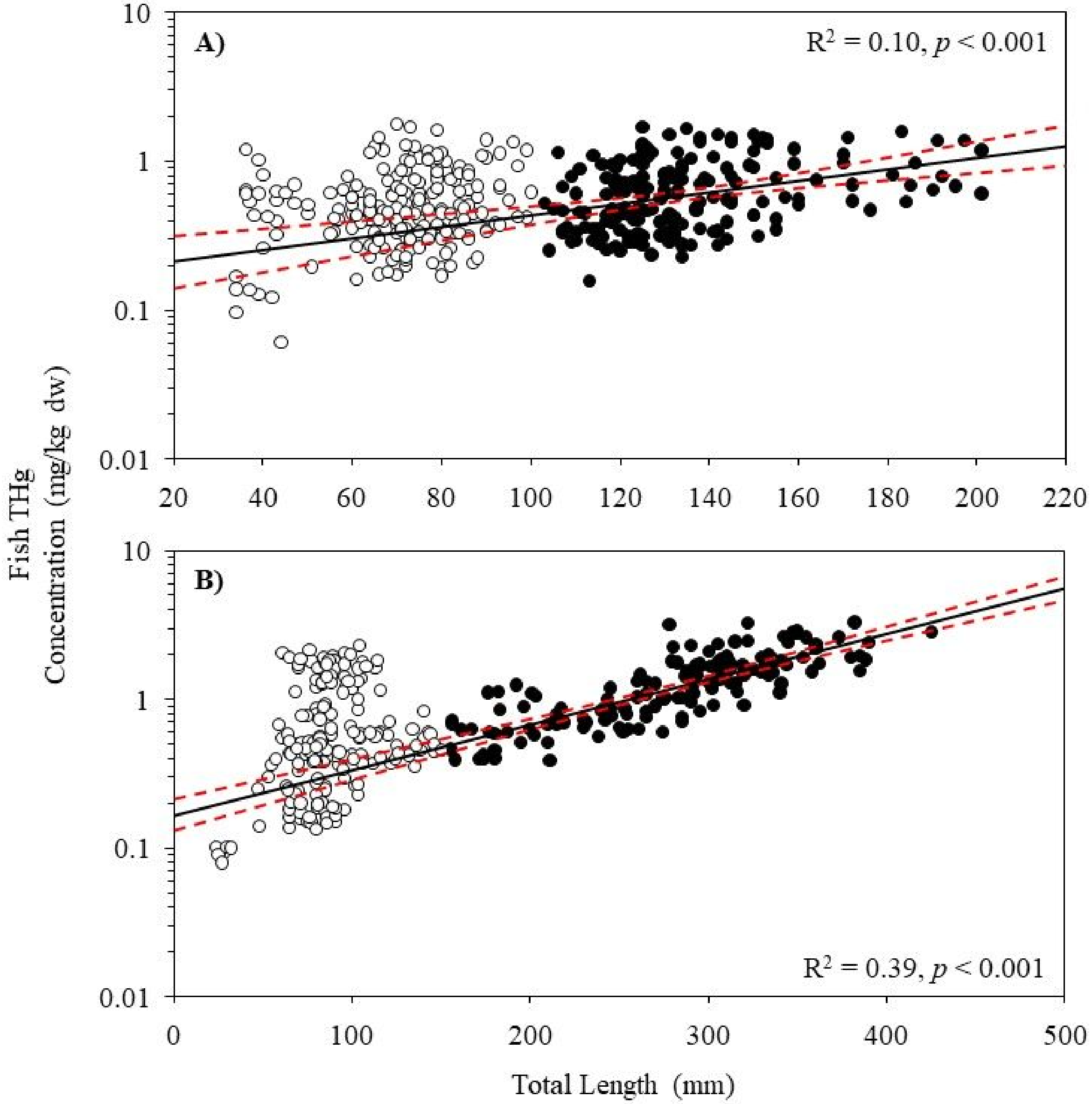

Figure S3: Relationships between total mercury (THg) concentrations and total length in **A)** Bluegill and **B**) Smallmouth Bass from Brownlee Reservoir between June 2018 and March 2020. Open points represent putative juveniles and closed points represent adult individuals.

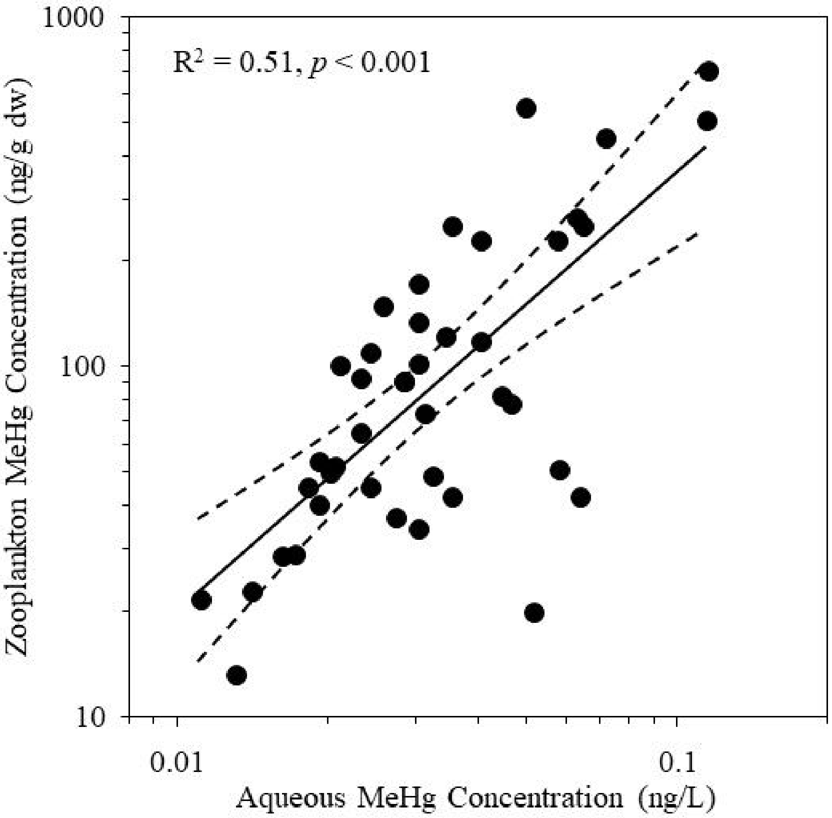

Figure S4: Relationship between methylmercury (MeHg) concentrations in filter-passing water and zooplankton from Brownlee Reservoir between June 2018 and March 2020.
